# Hypoxia Mediates Differential Response to Anti-EGFR Therapy in HNSCC Cells

**DOI:** 10.3390/ijms18050943

**Published:** 2017-04-29

**Authors:** Emilia Wiechec, Katharina Tiefenböck Hansson, Lisa Alexandersson, Jan-Ingvar Jönsson, Karin Roberg

**Affiliations:** 1 Department of Clinical and Experimental Medicine, Division of Cell Biology, Linköping University, Linköping 581 85, Sweden; jan-ingvar.jonsson@liu.se (J.-I.J.); karin.roberg@liu.se (K.R.); 2 Department of ENT—Head and Neck Surgery, County Council of Östergötland, Linköping 581 85, Sweden; katharina.tiefenbock@regionostergotland.se (K.T.H.); lisa.alexandersson@regionostergotland.se (L.A.); 3 Linköping Integrative Regenerative Medicine Centre, Linköping University, Linköping 581 85, Sweden; 4 Division of Oto-Rhino-Laryngology and Head & Neck Surgery, Linköping 581 85, Sweden

**Keywords:** hypoxia, HIF-1α, epithelial-mesenchymal transition (EMT), cancer stem cells (CSC), head and neck tumors, radiotherapy, cetuximab, cisplatin

## Abstract

Despite advances in the head and neck squamous cell carcinoma (HNSCC) treatment modalities, drug resistance and cancer recurrence are often reported. Hypoxia signaling through hypoxia-inducible factor 1 (HIF-1) promotes angiogenesis and metastasis by inducing epithelial-mesenchymal-transition (EMT). The aim of this study was to evaluate the impact of hypoxia on response to therapy as well as EMT and expression of stem cell markers in HNSCC cells. Five HNSCC cell lines (UT-SCC-2, UT-SCC-14, LK0412, LK0827, and LK0923) were selected for this study. The treatment sensitivity for radiation, cisplatin, cetuximab, and dasatinib was assessed by crystal violet assay. Gene expression of EMT and cancer stem cell (CSC) markers as well as protein level of EGFR signaling molecules were analyzed by qPCR and western blotting, respectively. Unlike UT-SCC-14 and LK0827, the LK0412 cell line became significantly more sensitive to cetuximab in hypoxic conditions. This cetuximab sensitivity was efficiently reversed after suppression of HIF-1α with siRNA. Additionally, hypoxia-induced EMT and expression of stem cell markers in HNSCC cells was partially revoked by treatment with cetuximab or knockdown of HIF-1α. In summary, our study shows that hypoxia might have a positive influence on the anti-EGFR therapy effectiveness in HNSCC. However, due to heterogeneity of HNSCC lesions, targeting HIF-1α may not be sufficient to mediate such a response. Further studies identifying a trait of hypoxia-specific response to cetuximab in HNSCC are advisable.

## 1. Introduction

Head and Neck Squamous Cell Carcinoma (HNSCC) is the sixth most common cancer worldwide, with over half a million new cases annually. The modern treatment is based on a multimodality strategy involving mainly surgery and radiotherapy. Additional treatments such as cisplatin-based chemotherapy or molecular targeted drugs like cetuximab have been developed during recent years, but are only provided to patients with advanced disease. Despite new treatment possibilities the five-year overall survival is still around 60% and treatment resistance as well as tumor recurrences remain a big problem.

Hypoxia has been shown to negatively influence the treatment outcome in many solid tumors, and this is also the case in HNSCC [1]. It arises in some areas of the tumor due to rapid tumor growth, deficient angiogenesis, vascular disturbances, and metabolic changes [2].

HIF (hypoxia-inducible factor) is a transcription factor that responds to oxidative stress. It is a heterodimer that consists of the oxygen-regulated α-subunit (with its three isoforms HIF-1α, HIF-2α, and HIF-3α) and the constitutively expressed β-subunit. Under normoxic conditions HIF-1α undergoes ubiquitylation by the von Hippel-Lindau protein leading to proteasomal degradation [3]. Under hypoxia HIF-1α is stabilized and is translocated into the nucleus resulting in angiogenesis, cell proliferation and survival, changes in the glucose metabolism, and migration, as well as resistance to chemo- and radiotherapy [4].

Epithelial-mesenchymal transition (EMT) is an important part in the process of tumor progression and metastasis. EMT is characterized by changes in cell-shape akin to mesenchymal cells, recognized by increased cell motility, loss of cell adhesion, and production of proteinases. This process includes loss of epithelial markers such as E-cadherin, a transmembrane glycoprotein [5,6], and elevated expression of mesenchymal markers such as vimentin (Vim), N-cadherin, fibronectin, snail, slug, Fox, and Twist [7]. Cells with EMT characteristics are found in increased numbers in tumors under hypoxic conditions [8,9].

In many solid tumors there are a small subset of cells that are involved in tumor invasion and metastasis. They are cancer-initiating cells, recognized by their cell surface phenotype, including surface markers such as high CD44 [7]. The theory of cancer stem cells (CSC) is now widely accepted, and was first shown in breast cancer cells [10]. CSC are now increasingly recognized to play a role in the metastasis in several types of cancer including HNSCC. Studies have shown that EMT contributes to cancer metastasis, and CSC frequently display EMT properties [11]. However, it is still unknown how hypoxia, CSC and EMT are interacting at a molecular level; thus there is need for further investigation. Finding therapeutic targets to eradicate the drug resistant CSC is most likely a key to reduce recurrence.

The aim of this study was to investigate the impact of hypoxia on the treatment response, EMT profile, and expression of stem cell markers in HNSCC.

## 2. Results

### 2.1. Hypoxia Induces Changes in the Treatment Response in HNSCC

**Figure 1:**
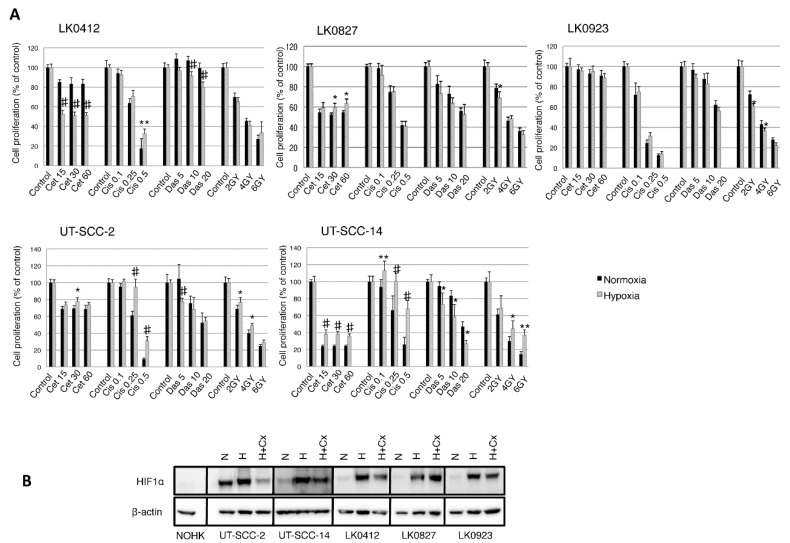
Hypoxia-induced treatment response in Head and Neck Squamous Cell Carcinoma (HNSCC) cell lines. Cell lines were cultured in normoxia and hypoxia. (**A**) After 24 h, they were treated with various concentrations of cetuximab (cet; nM), cisplatin (cis; µg/mL), dasatinib (das; nM), and radiation (2, 4, 6 Gy). After 9 days the cytotoxic/cytostatic effect on cell proliferation was determined by crystal violet assay. Cell proliferation is presented as the percentage of the untreated controls (mean values ± SD; *n* = 3, triplicates). For statistical analysis, one-way ANOVA with *post-hoc* Bonferroni analysis was used (* *p* < 0.05; ** *p* < 0.01; # *p* < 0.001); (**B**) Western blot analysis of hypoxia-inducible factor (HIF)-1α expression in normal oral human keratinocytes (NOHK) as well as UT-SCC-2, UT-SCC-14, LK0412, LK0827, and LK0923 HNSCC cells. Hypoxic cells were exposed to cetuximab (60 nM) for 3 days prior to harvesting for Western blotting; β-actin was used as the loading control. Abbreviations: N, normoxia; H, hypoxia; H + Cx, hypoxia in the presence of cetuximab; Cx, cetuximab.

We have previously screened a large number of HNSCC cell lines to determine their treatment sensitivities and phenotypes [12,13,14,15]. Based on these data, five cell lines, of which three (UT-SCC-2, UT-SCC-14, and LK0412) present an epithelial phenotype as well as two (LK0827 and LK0923) that resemble an EMT-phenotype [16] were selected for this study. To investigate the effect of hypoxia on the treatment sensitivity, the HNSCC cells were exposed to cisplatin (0.1, 0.25, and 0.5 µg/mL), cetuximab (15, 30, and 60 nM), dasatinib (5, 10, and 20 nM), or ionizing γ-irradiation (2, 4, and 6 Gy). In our settings, the number of surviving cells after exposure to chemotherapeutic drugs/ionizing γ-irradiation under normoxia and hypoxia was determined by crystal violet staining. Treatment with cetuximab in the absence of oxygen was associated with enhanced cell survival in the UT-SCC-2 and UT-SCC-14 cells exhibiting epithelial-like features in contrast to the LK0412 cells, which acquired sensitivity towards cetuximab when cultured in such conditions. Interestingly, hypoxia moderately increased sensitivity of the UT-SCC-14 and LK0412 cells to dasatinib. Furthermore, only minor differences in the hypoxia-driven treatment response were observed in the two cell lines possessing mesenchymal phenotype (LK0827 and LK0923; Figure 1A).

We further investigated the effect of cetuximab on the HIF-1α level during hypoxia. The hypoxia-mediated protein level of HIF-1α was reduced in cells treated with cetuximab with the highest inhibitory effect of cetuximab in UT-SCC-2 cells. However, we did not observe any cetuximab-mediated HIF-1α downregulation in the LK0827 and LK0923 cell lines. Interestingly, UT-SCC-2 cell line displayed a relatively high level of HIF-1α expression under normoxic conditions (Figure 1B).

### 2.2. Hypoxia-Induced mRNA Expression of the EMT and CSC Markers in HNSCC

To further explore whether hypoxia mediates EMT in HNSCC, the mRNA expression levels of E-cadherin, N-cadherin, vimentin, fibronectin, Twist1, and Foxc2 were analyzed by RT-qPCR.

**Figure 2:**
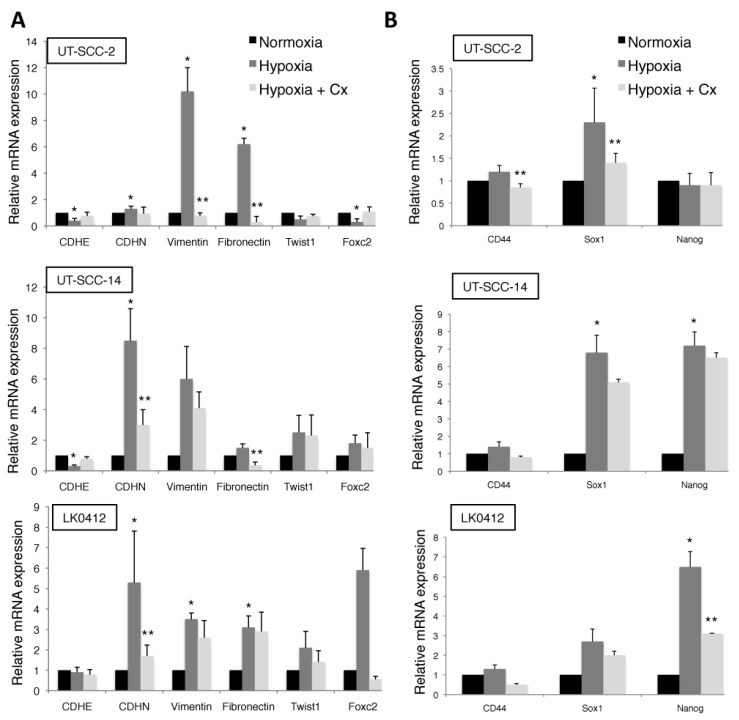
Hypoxia-induced epithelial-mesenchymal transition (EMT) and expression of stem cell markers in HNSCC. RT-qPCR was performed to analyze mRNA expression levels of EMT (**A**) and stem cell (**B**) markers in HNSCC cells following exposure to normoxic and hypoxic conditions for 7 days in the presence or absence of cetuximab (60 nM). The relative amount of analyzed genes is calculated using the 2^−ΔΔ*C*t^ method and amplification of GAPDH and β-actin were used as an internal standard. Data were normalized to cells cultured in normoxic conditions in each column graph (mean values ± SD; *n* = 3). * *p* < 0.05 versus N (normoxia) and ** *p* < 0.05 versus H (hypoxia) according to Student’s *t*-test.

As shown in Figure 2A, expression of EMT markers in analyzed cell lines was highly dependent on hypoxic conditions. In general, significantly increased levels of N-cadherin, vimentin, and fibronectin were observed under hypoxic conditions. Moreover, hypoxia-dependent EMT is associated with increases in the mRNA expression of the stem cell transcription factors, Sox1, and Nanog (Figure 2B). This pattern of hypoxia-induced EMT and expression of stem cell markers in HNSCC was not significantly affected by treatment with cetuximab (Figure 2A,B).

### 2.3. Effect of HIF-1α Downregulation on Treatment Response and mRNA Expression of EMT and Cell Stem Cell Markers in HNSCC Cells

To examine the involvement of HIF-1α in the hypoxia-induced drug resistance/sensitivity, EMT and expression of stem cell markers in HNSCC, the downregulation of HIF-1α with HIF-1α-specific siRNA was performed. The above described UT-SCC-14 (less sensitive to cetuximab under hypoxia) and LK0412 (sensitive to cetuximab under hypoxia) HNSCC cell lines were chosen for the analysis.

**Figure 3:**
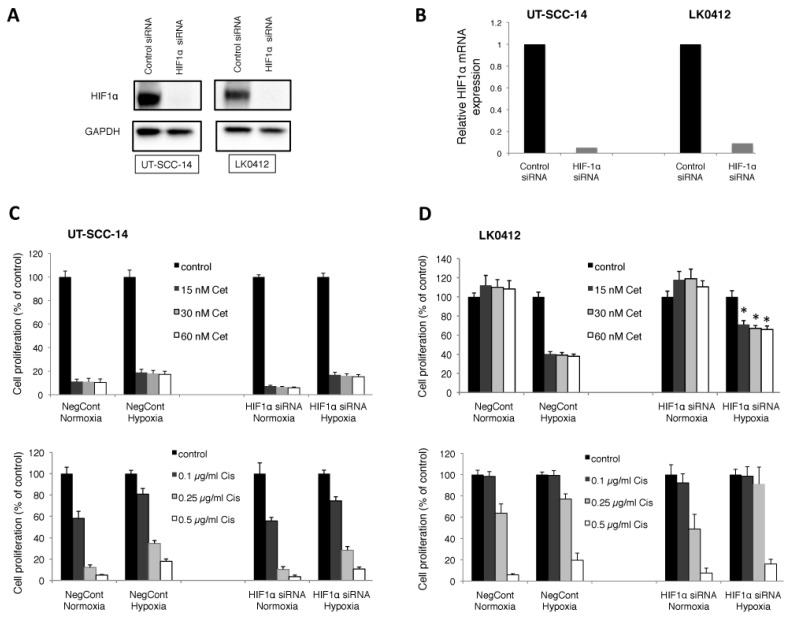
Effect of HIF-1α downregulation on drug response in HNSCC. The UT-SCC-14 and LK0412 cell lines were transiently transfected with either non-targeting siRNA (control siRNA) or HIF-1α-specific siRNA. The protein expression of HIF-1α was assessed by western blotting (**A**) and the mRNA expression level of HIF-1α was assessed by RT-qPCR (**B**) after 72 h of incubation in hypoxic conditions. (**C**,**D**) Cells were treated with various concentrations of cetuximab (cet; nM) and cisplatin (cis; µg/mL) 24 h post-transfection with either non-targeting siRNA (control siRNA) or HIF-1α-specific siRNA followed by exposure to hypoxic conditions. After 9 days the cytotoxic/cytostatic effect on cell proliferation was determined by crystal violet assay. Cell proliferation is presented as the percentage of the untreated controls (mean values ± SD; *n* = 3, triplicates). For statistical analysis, one-way ANOVA with post-hoc Bonferroni analysis was used (* *p* < 0.05).

We were able to reduce the HIF-1α protein level (Figure 3A) as well as HIF-1α mRNA (Figure 3B) by ≥80% compared to the cells treated with non-targeting, control siRNA. Knockdown of HIF-1α did not significantly improve sensitivity of the UT-SCC-14 cell line to either the cisplatin or the cetuximab treatment (Figure 3C). Unlike UT-SCC-14, cetuximab sensitivity was efficiently reversed in the LK0412 cell line upon suppression of HIF-1α (Figure 3D).

**Figure 4:**
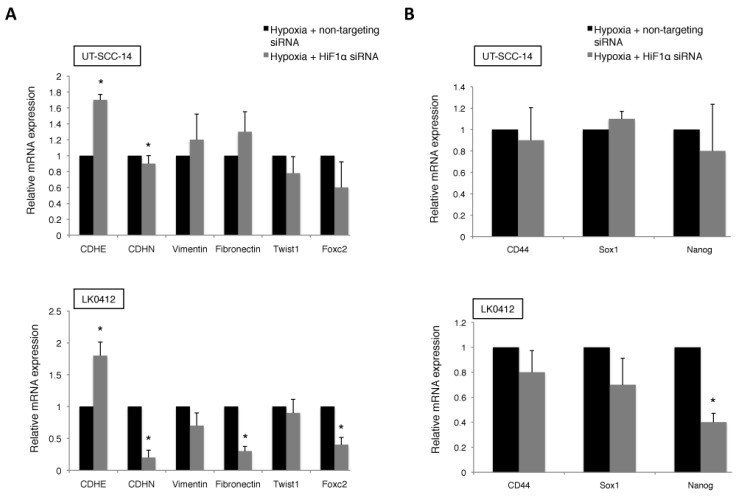
Effect of HIF-1α downregulation on EMT profile and expression of stem cell markers in HNSCC. The UT-SCC-14 and LK0412 cells were transiently transfected with either non-targeting siRNA or HIF-1α-specific siRNA and maintained under hypoxia for 72 h. The mRNA expression levels of (**A**) EMT markers and (**B**) stem cell markers in HNSCC cells cultured under hypoxia were analyzed by RT-qPCR. The relative amount of analyzed genes is calculated using the 2^–ΔΔ*C*t^ method and amplification of GAPDH and β-actin were used as an internal control. Data were normalized to cells cultured in hypoxic conditions and transfected with control, non-targeting siRNA in each column graph (mean values ± SEM; *n* = 3). * *p *< 0.05 according to Student’s *t*-test.

Moreover, suppression of HIF-1α with siRNA revoked the hypoxia-induced E-cadherin downregulation accompanied by downregulation of N-cadherin, fibronectin, and Foxc2 in LK0412 cell line when compared to a moderate effect in UT-SCC-14 cells (Figure 4A). Knockdown of HIF-1α did not have impact on mRNA levels of stem cell-specific markers in analyzed HNSCC cells (Figure 4B).

### 2.4. The Effect of Hypoxia on EGFR Downstream Signalling in Cetuximab Treated HNSCC Cells

**Figure 5:**
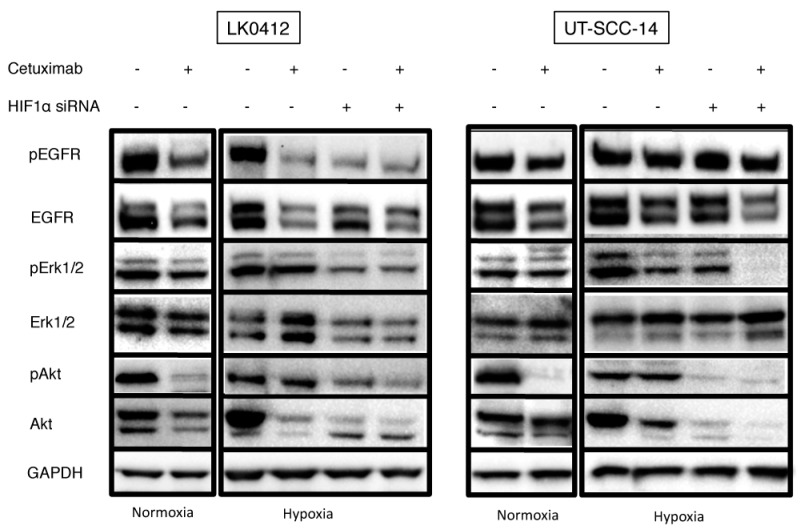
Effect of hypoxia on EGFR downstream signaling in HNSCC cell lines. Western blot analysis of the EGFR signaling pathway in LK042 and UT-SCC-14 HNSCC cells treated with 60 nM of cetuximab for 3 days; GAPDH was used as the loading control. Abbreviations: N, normoxia; N + Cx, normoxia in the presence of cetuximab; H, hypoxia; H + Cx, hypoxia in the presence of cetuximab; Cx, cetuximab.

The EGFR signaling pathway has been widely described to play a role in the pathogenesis of various cancer types including HNSCC. In this study, we focused on the impact of cetuximab on the EGFR signaling molecules (pEGFR, pAkt, pErk1/2) under hypoxic conditions. The UT-SCC-14 and LK0412 HNSCC cell lines exhibiting reduced (UT-SCC-14) or enhanced (LK0412) response to cetuximab in hypoxic conditions were studied. Both cell lines responded to cetuximab treatment by a decrease of pEGFR and EGFR expression irrespective of oxygen accessibility. However, cetuximab-mediated downregulation of pEGFR under hypoxia was more effective in the LK0412 cell line, which exhibits higher sensitivity to cetuximab in reduced oxygen conditions. Downregulation of pEGFR and EGFR was associated with reduced activation of pAkt in normoxic conditions while the effect of cetuximab on the level of pErk1/2 was comparable under normoxia and hypoxia in the analyzed cell lines. The combination of HIF-1α knockdown with cetuximab treatment was more effective against the PI3K/AKT signaling pathway inactivation than either treatment alone in the analyzed cells (Figure 5).

## 3. Discussion

The connection between CSC and EMT has become more evident over the last couple of years. It has been described that induction of EMT in mammary epithelial cells resulted in cells gaining stem cell properties while in HNSCC stem cell-like cells obtained from spheroid cultures exhibited an EMT phenotype with elevated levels of vimentin and α-smooth muscle actin [17]. During the last few years, hypoxia has become increasingly related to both EMT and the CSC phenotype including HNSCC [18,19,20]. HIF-1α induced by hypoxia has been found to promote upregulation of Snail and to attenuate the expression of E-cadherin, leading to EMT and increased cancer aggressiveness [21]. Hypoxia and induction of HIF-1α have also been shown to expand the subpopulation of cancer stem-like cells and to increase resistance to therapies in various cancer types [22,23,24]. Although a direct involvement of increased expression of HIF-1α under hypoxia is understandable, we have observed a relatively high basal level of HIF-1α in one of the analyzed HNSCC cell lines. A similar phenomenon has recently been described in prostate cancer, which presents a mechanism of HIF-1α stabilization by beta-arrestin1 (ARRB1) under normoxic conditions. This ARRB1-mediated “pseudohypoxia” is considered to confer a growth advantage of cancer cells and their adaptation to the rough environment observed in solid tumors [25,26]. Moreover, it has been reported that HIF-2α may regulate the expression of Sox-2, Oct4, and Nanog, and in glioma cells HIF-2α expression was observed only in the CSC population [27].

In this study, we have shown that hypoxia leads to mRNA increases of EMT-associated markers and stem cell transcription factors in HNSCC cells. We observed a cetuximab-induced downregulation of HIF-1α under hypoxic environment in three HNSCC cell lines with epithelial-like characteristics in contrast to two cell lines with mesenchymal phenotype. It has been proposed that cancer cells possessing mutations within the PTEN gene are more resistant to cetuximab-mediated reduction of the HIF-1α level [28], which might be of importance in our observations. Furthermore, the example of the LK0412 cell line provides evidence of hypoxia capability to sensitize cancer cells towards cetuximab treatment. Interestingly, knockdown of endogenous HIF-1α had the opposite effect on cetuximab-induced proliferative capacity of hypoxic LK0412 HNSCC cells, stressing its role in this process. Although a similar pattern of hypoxia-mediated sensitization to erlotinib has recently also been shown in HNSCC [29], the observed contribution of HIF-1α seems to be questionable. So far, the sensitizing effect of hypoxia towards cetuximab treatment was observed in one out of five analyzed HSCC cell lines. It is therefore advisable to launch a regular screening project to see how prevalent the observed effect is and to exclude the potential off-target effect of siRNA.

Furthermore, we have observed preferential toxicity of dasatinib towards hypoxic UT-SCC-14 and LK0412 cells. This suggests that a tyrosine kinase inhibitor (TKi) such as dasatinib displays increased potency against HNSCC cells under hypoxia probably due to an increased EMT phenotype. A similar effect of sensitivity to dasatinib in hypoxia was presented in MDA-MB-231 cells [30]. In addition, dasatinib did not exhibit a dose dependent effect in LK0412 cell line under normoxia, which suggests that hypoxia is not a predominant cause of resistance to therapy in HNSCC. A recent study on HNSCC cell lines has demonstrated that EGFR activation was a determinant of dasatinib resistance [31].

Interestingly, UT-SCC-2 and UT-SCC-14 cells cultured in hypoxia presented increased resistance to radiotherapy, cisplatin, and cetuximab, which was connected with EMT induction and cell stem-like phenotype. In our previous study, HNSCC cells were characterized based on their cell surface expression of EGFR and CD44. The CD44^high^/EGFR^low^ population identified in the LK0827 and LK0923 HNSCC cell lines exhibited the EMT-like morphology, with high expression of EMT-associated genes and stem cell transcription factors. Moreover, these cells presented an increased resistance to radiotherapy, cisplatin, cetuximab, and gefitinib (an EGFR tyrosine kinase inhibitor) [16]. We could not observe an apparent relationship between hypoxia and resistance to drug treatment in the LK0827 and LK0923 HNSCC cell lines, which suggests that there are other mechanisms besides hypoxia responsible for drug resistance in HNSCC.

There is accumulating evidence that execution of the EMT program in malignant tumor cells contributes to both increased metastasis and treatment resistance. In several tumor types, such as breast, lung, ovarian, and prostate, EMT has been linked to radioresistance [32,33,34] and Holz and colleagues demonstrated that HNSCC cells resembling mesenchymal phenotype are less sensitive to irradiation [35]. In our settings, hypoxia was responsible for acquired radioresistance in the analyzed HNSCC cells. In addition, our previous work demonstrated that the mesenchymal marker FN1 is significantly upregulated in radioresistant cells [36].

Treatment of the hypoxic LK0412, UT-SCC-2, and UT-SCC-14 cells with cetuximab correlated with downregulation of the HIF-1α level. Nevertheless, neither cetuximab treatment nor knockdown of HIF-1α with siRNA was able to completely abolish the hypoxia-induced EMT and CSC phenotype in the analyzed HSCC cell lines. Our results suggest that inhibition of HIF-1α may not be sufficient to overcome the hypoxia-induced EMT and stem-like phenotype. This points at coexistence of other factors besides HIF-1α that may contribute to the malignant phenotype of HNSCC cells under hypoxia. It has been recently reported that some of the genes modified by cetuximab were related to cancer associated fibroblasts (CAFs) and EMT together with markers of embryologic pathways like NOTCH and Wnt [37]. A previous study in human colon carcinoma has explored pharmacodynamics of cetuximab, identifying cetuximab-mediated changes in proteins that were implicated in glucose metabolism as well as hypoxic regulators and vasculogenesis [38].

Additionally, we have shown in our previous study that high sensitivity to cetuximab in UT-SCC-14 cell lines was associated with cetuximab-mediated reduction in pEGFR both in vivo and in vitro [39]. Nevertheless, neither inhibition of EGFR with cetuximab or HIF-1α knockdown could overcome the hypoxia-mediated decreased sensitivity to cetuximab in the UT-SCC-14 cell line compared to the LK0412 cell line. Considering the PI3K/AKT as a pro-survival signaling pathway, HIF-1α knockdown may inhibit activation of pAkt, a key player of this pathway. Unlike hypoxia, cetuximab treatment was efficient in reducing the pAkt expression level in normoxic conditions. Although a combination of cetuximab treatment with HIF-1α knockdown was more effective against PI3K/AKT pathway inactivation than single treatments, the hypoxia-mediated resistance of the UT-SCC-14 cells to cetuximab was observed. This specific response may be connected with tumor-specific mutations within K-Ras gene [40]. Furthermore, the LK0412 cell line exhibited higher sensitivity to cetuximab under hypoxia despite pAkt activation, which points at other mechanisms being responsible for such differential, hypoxia-mediated response to cetuximab in HNSCC.

In summary, our study shows that hypoxia might have a positive influence on the anti-EGFR therapy effectiveness in HNSCC. However, due to heterogeneity of HNSCC lesions, targeting HIF-1α might not be sufficient to improve a therapeutic effect of anti-EGFR treatment on hypoxic tumors. It is advisable to focus on hypoxia-specific response in order to find a mechanism by which hypoxia mediates sensitization towards cetuximab in HNSCC.

## 4. Materials and Methods

### 4.1. Cell Lines and Culture Conditions

In this study, two HNSCC cell lines, UT-SCC-2 and UT-SCC-14 from the University of Turku as well as three HNSCC cell lines, LK0412, LK0827, and LK0923 from the University of Linköping were used. All cell lines were derived from tissue specimens from patients diagnosed with HNSCC. All cell lines were cultured in Keratinocyte-SFM supplemented with antibiotics (penicillin 50 U/mL, streptomycin 50 µg/mL) and 1% FBS (all from GIBCO). The cells were given fresh culture media twice per week and were subcultured at confluence after detaching the cells with 0.25% trypsin + 0.02% EDTA at a weekly split ratio of approximately 1:2. Cultures in passages 10 to 25 were used in all experiments. Cells were screened periodically for mycoplasma contamination using DAPI staining and/or the Universal Mycoplasma Detection Kit (ATCC, Manassas, VA, USA).

### 4.2. Assessment of Intrinsic Radiosensitivity (IR), Intrinsic Cisplatin Sensitivity (ICS), and Intrinsic Cetuximab Sensitivity (ICmabS)

Tumor cells were seeded in 12-well plates (Corning, Corning, NY, USA) at densities of 300–800 cells/cm^2^ depending on the plating efficiency of each cell line. After 24 h, half of the cultures were moved to 1% O_2_ and the rest of the cultures were cultured under standard conditions (20% O_2_). After another 24 h, cetuximab (15, 30, and 60 nM; Erbitux^®^, Merck KGaA, Darmstadt, Germany), cisplatin (0.1, 0.25, 0.5 μg/mL) or dasatinib (5, 10, 20 nM; Sprycel^®^, Bristol-Myers Squibb, New York, USA) was added to selected cultures. Selected cells were irradiated (2, 4, or 6 Gy) with 4 MeV photons generated by a linear accelerator (Clinac 4/100, Varian, Palo Alto, CA, USA), delivering a dose-rate of 2.0 Gy/min. The cytostatic/cytotoxic effect was determined after another 9 days. After fixation in 4% paraformaldehyde (20 min), cells were stained with crystal violet (0.04% in 1% ethanol) for 20 min at room temperature and were then washed and air-dried. After solubilization in 1% SDS, the optical density at 550 nm was measured using a Victor plate reader (EG & G Wallac, Upplands Väsby, Sweden).

### 4.3. RT-qPCR

The RT-qPCR analysis was performed on a 7500 Fast Real-Time PCR system (Applied Biosystems, Stockholm, Sweden). Total RNA was extracted from the cells using the RNeasy Mini Kit (Qiagen, Sollentuna, Sweden), cDNA was synthesized using the High Capacity RNA-to-cDNA Kit (Applied Biosystems, Foster City, CA, USA), and FAM/MGB probes (Applied Biosystems, Foster City, CA, USA) were used for the PCR reaction. Amplification of two housekeeping genes, GAPDH and ß-actin was used as an internal standard. The comparative *C*_t_ method was applied to determine the fold-difference in expression levels relative to a control sample.

### 4.4. RNA Interference

Cells were seeded at a density of 12,000 cells/cm^2^ and were transfected 24 h later with HIF-1α mRNA-targeting siRNA or a non-targeting siRNA with no homology to any known human gene (AllStars Negative Control siRNA) with the HiPerFect transfection reagent (all from Qiagen). The final siRNA concentrations in the culture medium was 10 nmol/L. Twenty-four hours after transfection, the cells were sparsely seeded into 12-well plates (Corning, Corning, NY, USA). After another 24 h, half of the cultures were moved to 1% O_2_ and the rest of the cultures were cultured under standard conditions (20% O_2_), and after additional 24 h, cetuximab (Erbitux^®^; 15, 30, and 60 nM, Merck KGaA, Darmstadt, Germany), cisplatin (0.1, 0.25, and 0.5 µg/mL), or dasatinib (5, 10, and 20 nM) was added to selected cultures. The treated cells were grown for another 9 days, after which they were fixed and stained with crystal violet as described above. Knockdown was verified by quantitative real-time PCR and western blot after 72 h. A reduction in the mRNA level by at least 70% was achieved in all experiments.

### 4.5. Western Blot Analysis

Aliquots of protein (20 µg) were subjected to western blotting. The membranes were incubated with anti-HIF-1α (BD Biosciences, San Jose, CA, USA), anti-pEGFR (Millipore, Temecula, CA, USA), anti-EGFR (Santa Cruz Biotechnology, Dallas, TX, USA), anti-pErk1/2 (Cell Signaling Technology, Danvers, MA, USA), anti-Erk1/2 (Cell Signaling Technology, Danvers, MA, USA), anti-pAkt (R&D Systems, Minneapolis, MN, USA), and anti-Akt (R&D Systems, Minneapolis, MN, USA) overnight at 4 °C. After washing with TBS/0.1% Tween 20, the membranes were incubated with appropriate secondary antibody conjugated with horseradish peroxidase (Santa Cruz Biotechnology, Dallas, TX, USA). The bands were visualized with Western Blotting Luminol Reagent (Bio Rad, Hercules, CA, USA). Equal loading was verified by reprobing the membranes with an anti-ß-actin (Sigma Aldrich, St. Louis, MO, USA) or anti-GAPDH (BD Biosciences, San Jose, CA, USA) antibody.

### 4.6. Statistics

The data were analyzed using one-way ANOVA followed by Student´s *t*-tests with Bonferroni adjustment (IBM SPSS Statistics, version 21). *p*-values < 0.05 were considered significant.
